# Impact of Diabetes Mellitus Type 1 on Lebanese Families’ Quality of Life

**DOI:** 10.5005/jp-journals-10005-1486

**Published:** 2018-04-01

**Authors:** Balsam Noueiri, Nahla Nassif

**Affiliations:** 1 Associate Professor, Department of Pediatric Dentistry, Lebanese University Beirut, Lebanon; 2 Associate Professor, Department of Pediatric Dentistry, Lebanese University Beirut, Lebanon

**Keywords:** Diabetes mellitus type 1, Diabetic child, Family burden, Guilt feeling.

## Abstract

**Introduction:**

Diabetes mellitus type 1 (DM1) markedly alters the lives of individuals and their families. Family members can be affected by diabetes and its treatment, causing burden, distress, and reduced quality of life (QOL).

**Objective:**

The aim of this research is to study the relationship between the diabetic child and the family members, to evaluate the stress and emotional issues between siblings, and to weigh in on the psychological, affective, and financial burden that parents have to deal with in their daily life.

**Materials and methods:**

A total of 37 diabetic Lebanese families recruited from the Chronic Care Center (CCC) answered two questionnaires, one about general information and the other related to psychological and financial impact of DM1 and its oral complications on the families.

**Results:**

About 56.8% have monthly income below $1,000; 16.2% of parents have an educational upper limit of college degree; 83.8% of the mothers are housewives; 75.7% of parents feel guilty about their child’s condition; 89.2% feel that their diabetic child is frustrated with their diet. For 78.4%, the siblings are jealous of the diabetic child; 13.5% of parents are well aware of the oral complications of diabetes and 86.5% think that treating the diabetic child’s teeth is more important than the siblings’ ones; 91.9% assist their diabetic child’s toothbrushing, but 81.1% of family members do not visit the dentist regularly. A total of 100% allocate special budget for the diabetic child’s diet and 59.4% have an additional budget dedicated to the diabetic child’s treatment; 81.1% declared that their career is affected by their child’s illness.

**Conclusion:**

The diabetic child expressed frustration with their diet. The child’s siblings are jealous as they feel left behind. The parents experienced guilt feeling and psychological stress. They have social restriction and financial problems. The QOL of families living with a diabetic child is altered negatively.

**How to cite this article:** Noueiri B, Nassif N. Impact of Diabetes Mellitus Type 1 on Lebanese Families’ Quality of Life. Int J Clin Pediatr Dent 2018;11(2):61-65.

## INTRODUCTION

People with diabetes live in a society that does not understand their condition. To make the situation worse, only few people understand that diabetes is a condition that cannot be simply controlled by medication and that the presence of diabetes markedly alters the lives of individuals and their families.^1^

Williams et al^2^ reported that little is known about the individual and family factors associated with diabetes-specific family conflict. They examined whether background factors, diabetes variables, and psychological distress in parents and children and adolescents were associated with diabetes-specific family conflict. Furthermore, they studied the linkage between parental distress and family-level distress as it has not been examined fully in children and adolescents with diabetes. Their findings suggested a close link between psychological distress in parents and children and adolescents and reports of increased diabetes-specific family conflict.

Although family influence is perceived both positively and negatively, most people with diabetes welcome their support as it plays a crucial role in maintaining lifestyle changes and optimizing diabetes management. But diabetes and its treatment still affect the life of family members in several ways, causing, for example, different types of psychological distress. Family members can be adversely affected by diabetes and its treatment, causing burden, distress, and reduced QOL.^3-6^

As Burns et al^7^ found the relationship between QOL in individuals with diabetes and family members unclear, they conducted, in 2013, the first multinational study surveying adults with diabetes and their adult family members and health care providers. The results showed that diabetes has a substantial negative effect on family members of people with diabetes.

Although there are extensive studies that evaluate the medical, social, and psychological impact of diabetes on children, until now not many discuss the disease and its direct impact on family members, and even fewer investigate the financial issues in relation to diabetes and its oral complications.

The present research conducted on Lebanese families aims to study the relationship between the diabetic child and the family members, to evaluate the stress and emotional issues between siblings, and to weigh in on the psychological, affective, and financial burden that parents have to deal with in their daily life.

## MATERIALS AND METHODS

A cross-sectional approach was utilized to conduct this study. Our population of interest was 37 Lebanese diabetic children’s families from the CCC. The CCC is a Lebanese private nonlucrative institution with a multi-disciplinary medical team for preventing and monitoring certain chronic childhood diseases including DM1. With the help of one researcher, the parents answered two questionnaires. The first one consisted of 4 questions about general information related to socioeconomic factors, and the second one (13 questions) related to psychological and financial impact of DM1 and its oral complications on the families.

A consent form with a written approval was signed by the parents for their inclusion in the study.

The answers were scored on a three-point scale: 0 = never or not at all, 1 = moderately, and 2 = excessively.

The answers collected were then statistically analyzed using Statistical Package for the Social Sciences (version 18.0). The chi-square test was used to determine the level of association between variables. The level of significance was set at p < 0.05.

## RESULTS

Table 1 shows that 56.8% have monthly income below 1,000 USD, 40.5% between 1,000 and 1,500 USD, and 2.7% have above 1,500 USD; 35.2% of the fathers and 40.5% of the mothers have an educational level of primary degree; 48.6% of fathers, 43.3% of mothers reach the high school level and 16.2% of both parents have an educational upper limit of college degree; 83.8% of the mothers are housewives.

**Table 1:** **Table 1:** Financial and educational status of family members

		*Values (USD)*		*Proportions in %*		*p-value*	
Family monthly income		<1,000		56.8		0	
		1,000-1,500		40.5			
		>1,500		2.7			
Father’s education		Primary		35.2		0.053	
level		High school		48.6			
		College		16.2			
Mother’s education		Primary		40.5		0.085	
level		High school		43.3			
		College		16.2			
Does the mother work		No		83.8		0	
outside of the house?		Yes		16.2			

**Table 2:** **Table 2:** Impact of DM1 on the family members’ behavior

		*Values*		*Proportions in %*		*p-value*	
Is there any difference between		0		35.1		0.704	
raising a diabetic child and their		1		37.8			
nondiabetic siblings?		2		27.1			
Do you feel guilty about the		0		24.3		0.368	
diabetic child’s condition?		1		32.5			
		2		43.2			
Does the mother feel the need		0		2.7		0	
to be by her diabetic child’s side		1		21.6			
more than the norm?		2		75.7			
Do the siblings of the diabetic		0		21.6		0.027	
child feel any sort of jealousy		1		24.3			
toward him/her?		2		54.1			
Do you feel your diabetic child		0		2.7		0	
frustrated when their siblings are		1		8.1			
eating sweets?		2		89.2			
Are you aware of the oral		0		54.1		0.01	
complications of diabetes?		1		32.4			
		2		13.5			
Do you think that treating your		0		13.5		0	
diabetic child’s teeth is more		1		18.9			
important than treating his/her sibling’s teeth?		2		67.6			
Do family members visit the		0		81.1		0	
dentist regularly?		1		10.9			
		2		8			
Does the mother assist her		0		8.1		0	
diabetic child’s teethbrushing		1		21.6			
more than that of her other children?		2		70.3			
Do you allocate a special budget		0		35.1		0.469	
for the diabetic child’s oral		1		40.5			
health?		2		24.4			
Is there an additional budget		0		40.6		0.012	
allocated to the diabetic child’s		1		48.6			
treatment?		2		10.8			
Do you allocate a special budget		0		0		0	
for the diabetic child’s diet?		1		16.2			
		2		83.8			
Does the diabetic child’s		0		18.9		0.085	
condition affect your career?		1		48.6			
		_2_		_32.5_			

In Table 2, for 35.1% of parents, there is no difference between raising a diabetic child and their other children. However, 75.7% of parents feel guilty about their child’s condition and 97.3% of mothers need to stay by the side of the diabetic child more than the norm; 89.2% of parents feel that their diabetic child is frustrated when his siblings eat sweets and adversely 78.4% of parents testified that the siblings are jealous of the diabetic child.

A total of 13.5% of parents are well aware of the oral complications of diabetes and 86.5% think that treating the diabetic child teeth is more important than the siblings’ ones. Moreover, 91.9% of parents assist their diabetic child’s teethbrushing but 81.1% of families’ members do not visit the dentist regularly.

A total of 100% of parents allocate special budget for the diabetic child’s diet and 59.4% have an additional budget dedicated to the diabetic child’s treatment. The career of only 18.9% of parents is not affected by their child’s illness.

## DISCUSSION

The present study shows that few families have a monthly income over 1500 USD, the majority of both fathers and mothers interrupt their education before reaching college, and most of the mothers are housewives (Table 1). The latter reflects the modest socioeconomic level of our sample, which flows logically by their belonging to the nonlucrative institution, the CCC.

The treatment of DM1 is multifactorial, implicating the active participation of the whole family.^8^ In the same spirit, Correia Junior et al^9^ declared that DM interferes not only in the child but also in the family and social group, imposing profound modification in the lifestyle. Moreover, Williams et al^2^ declared that pediatric type 1 diabetes is often characterized as a “family disease” because of the important role of family interactions and parental support.

Almost two-thirds of parents admitted that they treat their diabetic child differently from their other children, and 97.3% of mothers feel the need to stay by their diabetic child’s side more than the norm (Table 2). It can be due to the fact that parents need to keep their child under supervision in order to control the glycemia in fear of its complications. According to Martins et al,^10^ the diabetic child’s mother fears the possibility of the occurrence of hypoglycemia when she is not around. Bennadi and Reddy,^11^ in their study about oral health-related QOL, mentioned that research on QOL has gained interest and visibility in recent decades, internationally. “How” we live and not just “how long” we live has increasingly become recognized as a central issue in health care and health research. They concluded that the perception of QOL has a subjective component and therefore, varies from one culture to another. The fact that the majority of mothers are housewives (Table 1) indicates that the care of their diabetic children falls back on them and requires their continuous presence around. Indeed, it is verified that DM interferes not only in the child but also in the family and social group, imposing profound modification in the lifestyle, and the main care is under the mother’s responsibility.^9^

Three-quarters of parents feel responsible of their child’s condition. This guilt feeling is common in all ethnicities and cultures. Cavini et al^12^ explained this feeling by the fact that mothers take responsibility for household care, so when the child falls ill, they blame themselves and feel despair which leads them to offset these feelings in the most intense care for children. Bowes et al^13^ mentioned that continuous feelings associated with grief, such as anger and guilt, were expressed by both fathers and mothers. In the same spirit, de Brito and Sadala^8^ reported that the parents punish themselves for the treatment and restriction imposed by the disease and end up following a protectionist policy regarding the child, which make them totally dependent.

The parents’ guilt feeling and special attention toward their diabetic child could create jealousy between siblings as indicated in the obtained results (Table 2). In fact, 78.4% of parents admitted that their healthy children feel jealous of their diabetic sibling. Manshaee et al^14^ declared that siblings can express jealousy toward their diabetic brother or sister. It might be explained by their young age, their possessive feeling toward their mothers, and their limited ability to cope with stress.

Although the solidarity between the family members is an essential part of the treatment of DM1 implicating the active participation of the whole family, not only the diabetic child,^8,15^ Correia Junior et al^9^ highlighted that the attention turned to the family’s diabetic member makes the other children of the couple feel left aside, and many times, they feel jealousy and injustice.

Vice versa, the diabetic child can express frustration and anger when they are deprived from sweets while their siblings are having some (Table 2). Considering the young age of the diabetic children in our sample (6-12 years old), it is understandable that they are not able to deal with their restricted diet; 97.3% of parents reported that their diabetic children can express nonsat-isfaction and anger. It can be interpreted by the fact that the diabetic child surrounded and supported continuously by the family cannot stand a situation of deprivation. Walker et al^16^ reported that food-related issues are universally identified as a source of frustration and it is openly acknowledged as a source of diabetes-specific conflict within homes.

Concerning the oral complications and their multifac-torial impacts on families, Alves et al^17^ stated that there is no doubt that patients need to be thoroughly informed about the risks of oral complications associated with the disease. In the present study, only 13.5% of parents are totally aware, 32.4% are partially aware, and 54.1% ignore the relation between DM1 and its oral complications. This poor awareness might be related to the parents’ low level of education as shown in Table 1. Valerio et al^18^ reported that lack of awareness and understanding of the relationship between diabetes and oral health may be an indicator of inadequate oral health literacy. A study in Portugal showed a significant relationship between low education of parents and poor glycemic control. Highly educated parents can be more oriented to the symptoms and complications of DM1 compared with less educated parents and it can be effective for better control of the disease.^19^ Another study in Kingdom of Saudi Arabia indicated that mothers with higher education had diabetic children with better glycemic control.^20^

A total of 86.5% of parents admit that they have tendency to prioritize the treatment of their diabetic child’s teeth more than that of their other children. It could be explained mostly by their guilt feeling (43.2%) and financial condition (56.8% < 1000 USD) as shown in Table 1.

Only 13.5% of parents consider that treating their nondiabetic children’s teeth is just as important as treating their diabetic child’s teeth and 81.1% of family members are not regular in their dental visits (p = 0.000). Those results could be explained by the fact that few parents are aware of general oral complications of DM1 (13.5%), the relatively high cost, the low income, and the fact that dental treatments are not covered by the social security in Lebanon.

A total of 91.9% of parents are present during their diabetic child’s teethbrushing. It might be explained by the parent’s guilt feeling and the continuous presence of the mother’s need to stay by the side of her diabetic child. Parents of children with DM1 perceive less cohesion and feel more anxiety and stress about parenting tasks compared with parents of healthy children.^21^ When DM is present in childhood, researches show the mother as the primary caregiver and her guidance is necessary once she takes care of her child.^10^ Correia Junior et al^9^ emphasized the importance of the mothers’ presence next to their diabetic child: they alter their activities for the welfare of the sick child, favoring the treatment and the assistance of the chronic disease once the transformation occasioned by the diagnosis of DM is inevitable.

The results on a special budget allocated to oral health were not statistically significant (p = 0.469).

On the contrary, parents reported that they assign special budgets for the treatment of diabetes (p = 0.012) and the required diet (p = 0.000). Even though the CCC provides their patients with insulin and blood glucose monitoring units, 10.8% of parents reported a struggle to cover the expenses of their child’s treatment and 83.8% for the special diet. It can be due to the low financial income of the majority of the families as shown in Table 1. Correa Junior et al^9^ highlighted the financial cost as a determinant factor for the family members to adhere to the treatment of a diabetic child, once the long-term treatment makes financial dynamic onerous, due to the acquisitions of flasks of insulin, glucometer, and its strips and the syringes to apply medication and expenditures with food.

About 81% of parents find that their child’s condition affects their career due to absence. They reported that it is essentially due to mandatory regular medical visits to the CCC and sometimes due to the sickness of their child. Mandic et al^22^ mentioned that caring for children with medical complexity frequently impacts employment and careers of caregivers and spouses. Employed and nonemployed caregivers invest substantial time in care.

Moreover, Martins et al^10^ considered mothers as the main person responsible for their children, as they abdicate their own appointments in order to assume the therapeutic procedures and glycemic control, living in function of the sickness of the child.

## CONCLUSION

The main findings of this study show that the diabetic child was properly supervised and his life was well framed inside the family. Despite the special attention, the diabetic child expressed frustration regarding their restricted diet. Diabetic child’s family members perceived many functional problems in their daily life. Parents experienced guilt feeling and psychological stress due to their child’s condition. Moreover, they expressed a social restriction and financial problems to cope with. The findings also pointed toward emotional issues perceived by the diabetic child’s siblings as they feel left behind. All these relevant results indicate that the QOL of families living with a diabetic child is altered negatively. In order to improve their well-being, it is very important to provide them more help by offering special psychological support to manage the difficulties of diabetes. Furthermore, low-income Lebanese families must receive financial aid from the Lebanese government or local nongovernmental organizations dedicated to improve the oral health of all members.
